# Novel Recombinant Norovirus in China

**DOI:** 10.3201/eid1205.051566

**Published:** 2006-05

**Authors:** Tung Gia Phan, Hainian Yan, Yan Li, Shoko Okitsu, Werner E.G. Müller, Hiroshi Ushijima

**Affiliations:** * The University of Tokyo, Tokyo, Japan;; † Kunming Medical College, Kunming, People’s Republic of China;; ‡ Universität Mainz, Mainz, Germany

**Keywords:** Recombination, China, norovirus

**To the Editor:** Norovirus (NoV), the distinct genus within the family *Caliciviridae*, is a major cause of sporadic cases and outbreaks of acute gastroenteritis in humans (*1*). NoV possesses a positive-sense, single-stranded RNA genome surrounded by an icosahedral capsid. The NoV genome contains 3 open reading frames (ORFs). ORF1 encodes nonstructural proteins, ORF 2 encodes capsid protein (VP1), and ORF3 encodes a small capsid protein (VP2). NoV is still uncultivable by standard culture with different cell lines. However, expression of either VP1 or both VP1 and VP2 with recombinant baculoviruses formed viruslike particles that are morphologically and antigenically similar to the native virion (*2*).

A fecal specimen was collected from an infant hospitalized with acute gastroenteritis in Kunming, China, in November 2004 and was tested for diarrheal viruses in a cooperative laboratory in Japan. The viral genome was extracted by using a Qiagen kit (Qiagen, Hilden, Germany). Polymerase chain reaction with specific primers resulted in the identification of astrovirus, rotavirus, sapovirus, adenovirus, and NoV genogroup I (GI) and GII (*3*). NoV polymerase was also amplified to identify recombinant NoV with primers Yuri22F and Yuri22R (*4*). Products were sequenced directly, and sequence analysis was performed by using ClustalX and SimPlot.

The fecal specimen was positive for NoV GII. The Figure shows that the 146/Kunming/04/China sequence clustered into the distinct GII genotype 7 (Leeds/90/UK cluster). 146/Kunming/04/China was classified into the Saitama U4 cluster (GI/6) when polymerase-based grouping was performed. Altogether, 146/Kunming/04/China was expected to be the recombinant NoV with GII/7 capsid and GII/6 polymerase.

**Figure:**
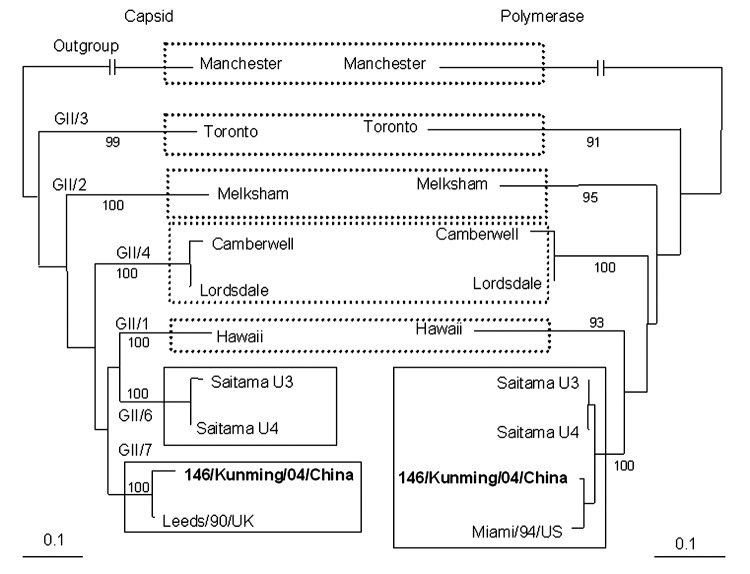
Changes in norovirus (NoV) genotypes on the basis of phylogentic trees of nucleotide sequences of 146/Kunming/04/China. Trees were constructed from partial nucleotide sequences of capsid and polymerase regions of 146/Kunming/04/China. 146/Kunming/04/China is **boldface**. Dashed boxes indicate the maintenance of genotypes of reference NoV strains, and solid boxes indicate the involvement of NoV genotypes with recombinant NoV 146/Kunming/04/China. A phylogenetic tree with 100 bootstrap resamples of the nucleotide alignment datasets was generated by using the neighbor-joining method with ClustalX. The genetic distance was calculated by using the Kimura 2-parameter method (PHYLIP). The scale indicates nucleotide substitutions per position. The numbers in the branches indicate the bootstrap values. Manchester strain was used as an outgroup strain for phylogenetic analysis. The nucleotide sequence of NoV strain 146/Kunming/04/China had been submitted to GenBank and has been assigned accession no. DQ304651. Reference NoV strains and accession nos. used in this study are as follows: Manchester (X86560), Toronto (U02030), Melksham (X81879), Camberwell (AF145896), Leeds/90/UK (AJ277608), Lordsdale (X86557), Hawaii (U07611), Saitama U3 (AB039776), Saitama U4 (AB039777), and Miami/94/US (AF414410).

To eliminate the possibility of co-infection with 2 different NoV genotypes, to localize the potential recombination site, and to clarify a possible recombination mechanism, the ORF1/ORF2 overlap and flanking polymerase and capsid regions of 146/Kunming/04/China was amplified with primers Yuri22F and GIISKR to produce a 1,158-bp amplicon (*3**,**4*). When the sequence of 146/Kunming/04/China was compared with that of Saitama U4 by using SimPlot, a recombination site was found at the ORF1/ORF2 overlap. Before this junction, 146/Kunming/04/China and Saitama U4 were homologous. After the ORF1/ORF2 overlap, however, the homology was notably different. SimPlot showed a sudden drop in the nucleotide identity for 146/Kunming/04/China. ClustalX showed that 146/Kunming/04/China shared a high identity (93%) in the polymerase region and a low identity (78%) in the capsid region with Saitama U4. In contrast, high identity (95%) in the capsid region was found between 146/Kunming/04/China and Leeds/90/UK. Since Leeds/90/UK polymerase was not available in GenBank, the polymerase homology between 146/Kunming/04/China and Leeds/90/UK was unknown. Polymerase of 146/Kunming/04/China was almost identical with that of Saitama U4, but the capsids of 146/Kunming/04/China and Leeds/90/UK were distinctly different from that of Saitama U4. This genetic pattern of 146/Kunming/04/China implied a novel, naturally occurring recombinant NoV with GII/7 capsid and GII/6 polymerase.

RNA recombination is a mechanism for virus evolution (*5*). Literature documenting recombination in NoV is fairly rich, but none is from China (*6*). Therefore, 146/Kunming/04/China was not only the first but also the first recombinant NoV from China. This isolate shared the closest sequences of polymerase and capsid with Saitama U4 and Leeds/90/UK, respectively. Strain Saitama U4 was detected in 1997 in Japan (*7*), whereas strain Leeds/90/UK was detected in 1990 in the United Kingdom (*8*). Quite possibly, Saitama U4 and Leeds/90/UK were parental strains of 146/Kunming/04/China. However, the distant geographic relationship of these strains obscured evidence of where and when the recombination event occurred. This phenomenon also suggested that these parent strains or this progeny strain might be more prevalent than is often assumed.

Recombination depends on various immunologic and intracellular constraints. Recombinant viruses are all alike in that they successfully pass through 5 stages: 1) successful co-infection of a single host, 2) successful co-infection of a single cell, 3) efficient replication of both parental strains, 4) template switching, and 5) purifying selection (*9*). In this study, 146/Kunming/04/China was recovered from a patient with diarrhea, fever, and vomiting. This observation indicated that this strain theoretically fulfilled all prerequisites for recombination.

The NoV capsid is predicted to be well suited for genotype classification (*10*). In this study, 146/Kunming/04/China belonged to 2 distinct genotypes, 7 and 6, by capsid- and polymerase-based groupings, respectively. Moreover, the recent demonstration of recombination in an increasing number of NoVs suggests that it is a more widespread event than was previously realized. Consequently, the phylogenetic classification of NoV on the basis of on capsid sequence is questionable. We suggest that classification of NoV strains should rely on not only capsid sequence but also polymerase sequence.

In conclusion, our results described the genetic characterization of novel, naturally occurring recombinant NoV and increased evidence for the worldwide distribution of recombinant NoV. This report is the first to describe acute gastroenteritis caused by recombinant NoV in China and warns of the threat it poses.
